# Weak Antilocalization and Anisotropic Magnetoresistance as a Probe of Surface States in Topological Bi_2_Te_x_Se_3−x_ Thin Films

**DOI:** 10.1038/s41598-020-61672-1

**Published:** 2020-03-16

**Authors:** Gregory M. Stephen, Owen. A. Vail, Jiwei Lu, William A. Beck, Patrick J. Taylor, Adam L. Friedman

**Affiliations:** 1 Laboratory for Physical Sciences, 8050 Greenmead Dr., College Park, MD 20740 United States; 2 Army Research Laboratory, 2800 Powder Mill Rd., Adelphi, MD 20783 United States; 3 Department of Materials Science and Engineering, University of Virginia, Charlottesville, VA 22904 United States

**Keywords:** Condensed-matter physics, Quantum physics, Physics, Electronics, photonics and device physics, Electronic and spintronic devices, Materials science, Materials for devices, Electronic devices

## Abstract

Topological materials, such as the quintessential topological insulators in the Bi_2_X_3_ family (X = O, S, Se, Te), are extremely promising for beyond Moore’s Law computing applications where alternative state variables and energy efficiency are prized. It is essential to understand how the topological nature of these materials changes with growth conditions and, more specifically, chalcogen content. In this study, we investigate the evolution of the magnetoresistance of Bi_2_Te_x_Se_3−x_ for varying chalcogen ratios and constant growth conditions as a function of both temperature and angle of applied field. The contribution of 2D and 3D weak antilocalization are investigated by utilizing the Tkachov-Hankiewicz model and Hakami-Larkin-Nagaoka models of magnetoconductance.

## Introduction

In topological insulators (TIs), a finite band gap in the bulk is accompanied by metallic surface states with linear dispersion, resulting from band inversion^1,2^. These surface states have a variety of potential applications owing to their robustness resulting from topological protection, potentially high mobilities, and spin-momentum locking, making them ideal candidates for spin-transport^3–8^. In particular, devices for applications in advanced computing and logic beyond Moore’s law have been envisioned^9–13^.

Bi_2_Se_3_ and Bi_2_Te_3_ are prototypical TIs that have been widely studied in order to understand this vibrant class of materials^14–19^. This is because they are relatively simple to grow as thin films^20–22^, and both possess a single Dirac point at accessible doping levels^23^. Despite the great amount of research into their topological nature, little work has been done to understand how changing concentrations with either Se or Te affect the overall topological properties. Insight into the nature of the conducting surface states as well as their robustness to chemical substitution is vital to effectively utilizing these materials. Further rationale for investigating Bi_2_Te_x_Se_3−x_ alloys is that they offer robust Dirac dispersion for surface conduction combined with a path toward reduced bulk conductivity compared to their parent compounds at either x = 0 or 3^24^. Specifically, the Bi_2_Te_2_Se alloy represents a special line compound whose bulk conductivity is sufficiently low that surface and bulk transport may be distinguished^25^. The interest, therefore, is to obtain high quality epitaxial thin films that facilitate transport mediated by topological surface states distinct from bulk electronic states. Moreover, in order to use these materials in the proposed applications, the material properties must be optimized.

In this work, we perform magnetoresistance (MR) measurements on four Bi-based topological material films: Bi_2_Se_3_, Bi_2_Te_3_, and two films with a mixture of Se and Te under constant growth conditions. We observe the 2D weak anti-localization (WAL) indicative of topological materials and we explore anisotropic magnetoresistance effects. We find a strong angle-independent WAL contribution in Bi_2_Se_3_ resulting from 3D bulk states. 2D states arise as Te replaces Se, creating a sinθ dependence to the WAL though also accompanied by an increasing classical MR background. However, these 2D states disappear in the pure Bi_2_Te_3_.

## Thin Film Growth and Device Fabrication

**Figure 1:**
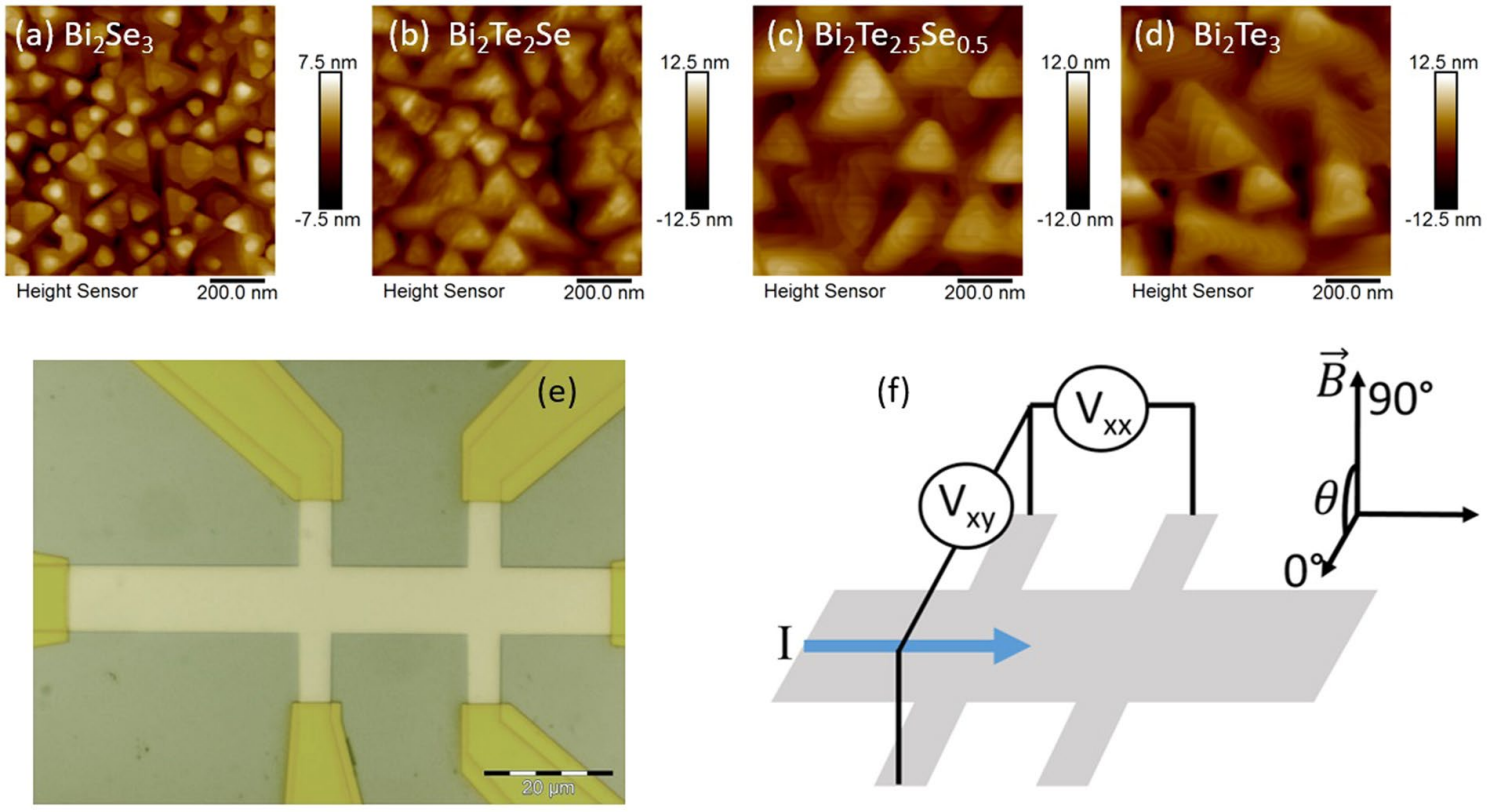
(**a–d**) AFM images of the four alloys used in this study. The surface height variation for these same films was between 15 nm for Bi_2_Se_3_ and 25 nm for Bi_2_Te_3_. (**e**) Optical image of one 10 × 20 µm hall bar. (**f**) Schematic of hall bar and magnetic field orientation.

MBE growth of Bi_2_Te_x_Se_3−x_ was performed on (001) semi-insulating GaAs. During MBE growth, the relative (Te + Se)/Bi beam-equivalent flux ratios for all epitaxial layers ranged from 15–20, and the nominal growth temperature was constant at 290 °C. For consistency, the thickness of all films was limited to 50 nm. The specific alloys that were obtained in this study include pure Bi_2_Se_3_ (x = 0), Bi_2_Te_2_Se_1_ (x = 2), Bi_2_Te_2.5_Se_0.5_ (x = 2.5) and pure Bi_2_Te_3_ (x = 3). X-ray, RHEED and TEM characterization confirmed high quality epitaxy throughout the alloy system with a sharp interface with the substrate. (see Supplemental Materials Figs. S5–7). Nonetheless, AFM analysis [Fig. 1(a–d)] of the highly specular films showed that the surface height varies between 15 nm for Bi_2_Se_3_ and 25 nm for Bi_2_Te_3_, similar to other MBE grown films in the literature^22,26,27^. This discrepancy is likely due to the highly polycrystalline nature of the films.

Mesoscopic Hall bars (10 × 20 µm^2^, Fig. 1(e)) were defined lithographically with an argon plasma etch. While plasma is known to cause damage, the surface was protected by a 1.5 μm thick, spun-on layer of poly(methyl methacrylate) (PMMA) such that the edges of the mesa were sharp, and similar measurements using similar un-patterned films with pressed indium contacts showed similar carrier densities. Electron beam evaporated gold (150 nm) with a titanium adhesion layer (10 nm) provided Ohmic contact to the Hall bar. Gold wire bonds were affixed with a ball bonder using indium spheres without heating or ultrasonic agitation.

## Magnetoresistance Measurements and Analysis

Magnetoresistance (MR) was measured for all four samples in a variable temperature cryostat set in a 1 T resistive magnet on a rotating platform. Sample measurement geometry was as shown in the schematic depicted in Fig. 1(f). For a majority of the measurements, the magnetic field is first applied in-plane with the film and perpendicular to the current direction, as indicated in the figure. The magnet is then incrementally rotated around the sample in the cryostat using the rotating platform such that the field is eventually out-of-plane with the sample. Other studies in the literature observed an anomalous negative magnetoresistance in Bi_2_Se_3_ when the magnetic field is both in-plane with the film and parallel to the applied current^28^. However, the negative component is expected to dominate at fields significantly higher than those used in our experiment. Nonetheless, we performed the measurement using this geometry on our samples as well and indeed observed similar results to the geometry shown in Fig. 1 (See Supplemental Materials, Fig. S1).

**Figure 2:**
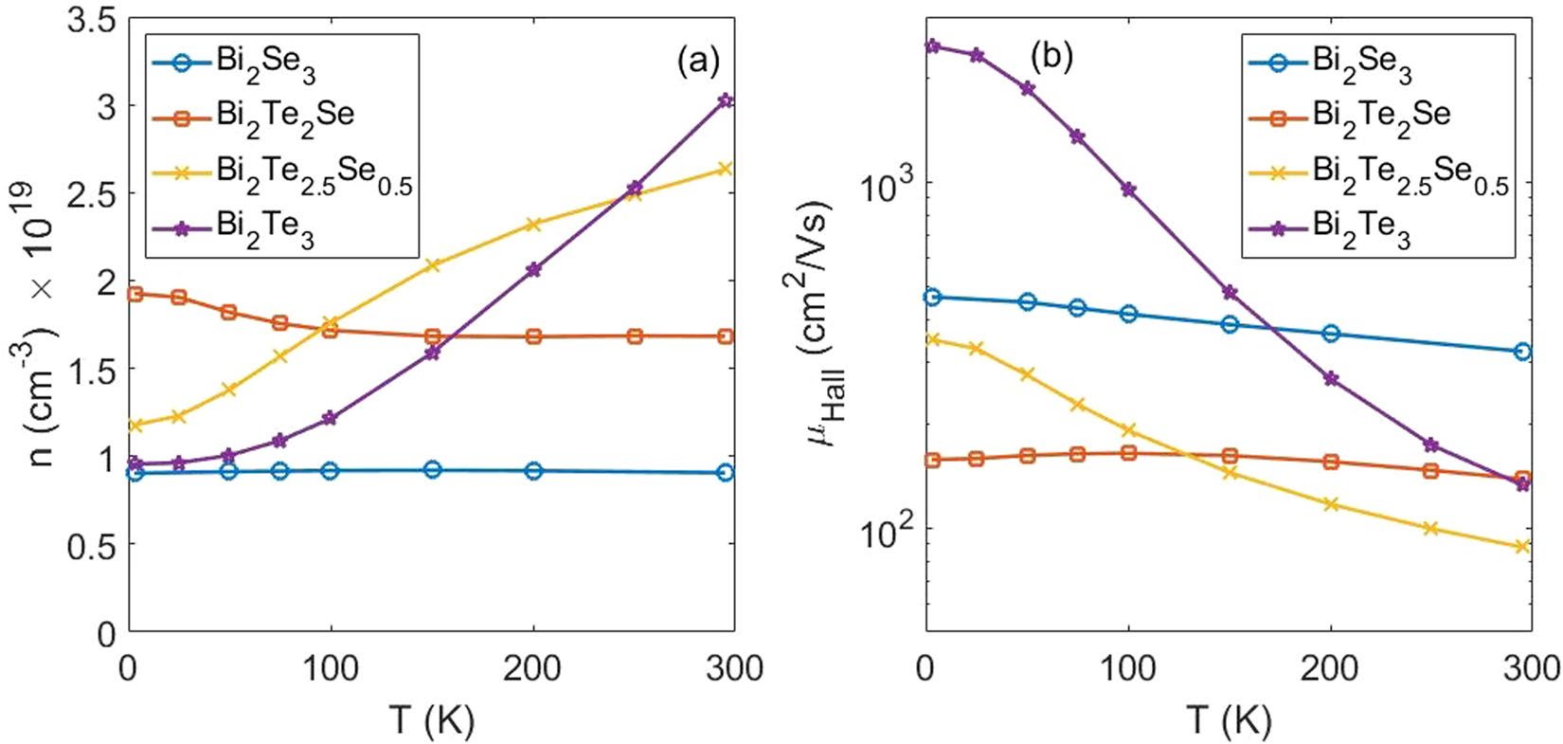
Carrier concentration (**a**) and mobility (**b**) versus temperature for the various Te concentrations. The large carrier concentration of 1019 cm-3 indicates these samples are heavily doped into the conduction band. However, the reduced mobility for the two intermediate samples suggests a stronger proportion of the conduction occurs in the surface. (See Supplemental Materials Fig. S8 for resistance versus temperature).

First, we measure mobility of all of the samples. Hall mobilities ${\mu }_{Hall}$ and carrier concentrations $n$ were determined using the ordinary Hall effect $d{R}_{xy}/dB\,=\,1/net$, where $t$ is the sample thickness, *e* is the elementary electronic charge, and ${\mu }_{Hall}=1/ne\rho$. The temperature dependence of the carrier concentrations and Hall mobilities are shown in Fig. 2. In Bi_2_Se_3_, *n* is nearly independent of temperature due to the semimetallic nature of the Dirac state, while *n* in Bi_2_Te_3_ has a strong exponential dependence due to the stronger influence of the bulk bandgap in the transport. Conversely, the mobility in Bi_2_Te_3_ and Bi_2_Te_2.5_Se_0.5_ decreases inversely proportional to T, while the mobility in Bi_2_Se_3_ and Bi_2_Te_2_Se changes less with T. The strength of the decrease in the mobility with temperature relates to the amount of scattering within the material. For topologically protected states, the scattering should be reduced, thus maintaining a more constant mobility with temperature. The increase (decrease) in carrier concentration (mobility) is consistent with the Bi_2_Te_3_ and Bi_2_Te_2.95_Se_0.05_ samples having conventional conducting states, while the steadier mobility in Bi_2_Se_3_ and Bi_2_Te_2_Se is strong evidence of topological protection. The anomalous slight increase in mobility for Bi_2_Te_2_Se could result from ionized impurity scattering^29,30^. Our measured mobilities of 100–1000 cm^2^/Vs correspond to mean free path on the order of 10 nm, corresponding to diffusive transport within our samples^6^.

**Figure 3:**
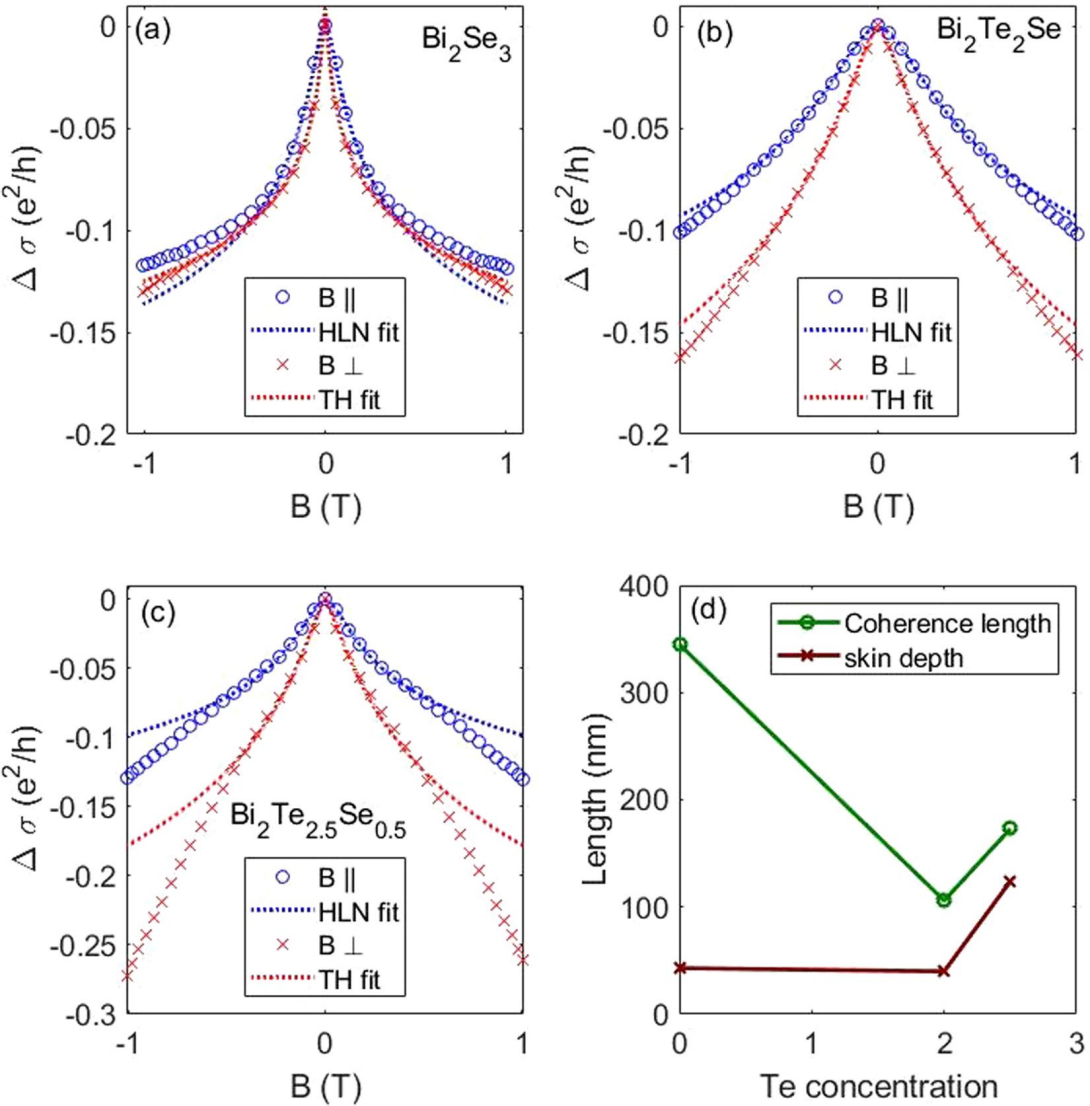
Magnetoconductance ($\Delta \sigma$) vs. Magnetic field for both in-plane and out-of-plane magnetic fields and the Hikami-Larkin-Nagaoka (HLN) and Tkachov-Hankiewicz (TH) fits with a linear background term (quadratic for x = 2.5). Fits at 3 K for the (**a**) Bi_2_Se_3_, (**b**) Bi_2_Te_2_Se, and (**c**) Bi_2_Te_2.5_Se_0.5_ samples for in-plane and out-of-plane fields. WAL was extremely weak compared to the much stronger quadratic background for Bi_2_Te_3_. Thus, it is not included in these plots. (**d**) Extracted coherence length from the HLN fit is used in the TH fit to calculate the skin depth.

Figure 3 shows Magnetoconductance ($\Delta \sigma$) vs. Magnetic field for both in-plane and out-of-plane magnetic field for the samples that contained Se. Open circles in blue show the data for the in-plane applied magnetic field, while the dashed lines of the same color show the fits to the models, discussed below. Likewise, red “x” marks show the data for the out-of-plane magnetic field and the red dashed line shows the fits. The pure Bi_2_Te_3_ sample show a very weak WAL cusp, however the strength of the quadratic background drastically increases the uncertainty in the fitting. Further information can be found in the Supplemental Materials Figs. S3, S4. At 3 K, weak antilocalization (WAL) is observed with a magnetic field applied both in-plane (0°) and out-of-plane (90°), persisting up to 50 K (see Supplemental Materials Fig. S3). For an out-of-plane magnetic field *B*, the Hikami-Larkin-Nagaoka model for 2D WAL in the strong spin-orbit coupling limit gives the change in conductance as

1a $$\frac{\Delta {\sigma }_{xx,\perp }}{{\sigma }_{0}}=\frac{\alpha {e}^{2}}{2\pi h}\left[\psi \left(\frac{{B}_{\perp }}{B}+\frac{1}{2}\right)-\,\mathrm{ln}\left(\frac{{B}_{\perp }}{B}\right)\right]$$

1b $${B}_{\perp }=\frac{\hslash }{2e{L}^{2}},$$

where $\psi$ is the Digamma function, $L$ is the coherence length, and *e* and *h* are the electron charge and Plank constant, respectively^31–33^. For Dirac states, the coefficient α is expected to be 1/2 for each Dirac cone^34^. As is evident in Fig. 3, the model fits the data well. From the fits, we extract the localization length and α. The 3 samples give values of α = 0.43, 0.50, and 0.42 for Bi_2_Se_3_, Bi_2_Te_2_Se, and Bi_2_Te_2,5_Se_0.5_, respectively. This range is consistent with literature and indicates 2D topological surface transport^14,34^.

A linear background is subtracted for the Bi_2_Se_3_ and Bi_2_Te_2_Se samples, and a quadratic background for the Bi_2_Te_2,5_Se_0.5_. The character of the backgrounds is derived from the MR behavior up to 9 T (see Supplemental Materials Fig. S4). Similar linear magnetoresistance has previously been observed in these materials, likely arising from the polycrystalline nature of the films, observed here in the AFM images in Fig. 1 ^35–39^. As evidenced by the magnetoresistance data, the quadratic contribution decreases as Te is replaced with Se. The normalization of using [R(B) − R(0)]/R(0) shows the relative contribution of the two component independent of the absolute resistance.

Unusually, TIs exhibit WAL with an applied in-plane field as well. This behavior can partially be explained by WAL originating from 3D states due to high spin-orbit coupling in the bulk, where the effect is independent of field angle^40^. Additionally an in-plane contribution for 2D states is hypothesized to arise due to a unique hexagonal warping of the surface state energy spectrum at all applied magnetic field angles. In a parallel magnetic field, the magnetic flux through the surface states decays exponentially up to a certain skin depth, defining an effective surface state depth. The magnetoconductance in a TI with an in-plane magnetic field is derived by Tkachov and Hankiewicz in ref. ^41^ to be

2a $$\frac{\Delta {\sigma }_{xx,\parallel }}{{\sigma }_{0}}=-\,\frac{{e}^{2}}{2\pi h}\,\mathrm{ln}(1+\frac{{B}^{2}}{{B}_{\parallel }^{2}})$$

2b $${B}_{\parallel }=\frac{2L}{\lambda }{B}_{\perp }=\frac{\hslash }{\lambda eL},$$

where, $\lambda$ is the effective surface state skin depth. For a purely 2D surface state ($\lambda =0)$, the argument of the log term reduces to 1, eliminating the change in magnetoconductance for an in-plane magnetic field. The existence of in-plane WAL requires a finite skin depth for the surface states, as well as a wide enough field range or small enough $\lambda /L$ such that the effect is observably non-quadratic. For sufficiently large values of $\lambda /L$, the in-plane magnetoconductance will be quadratic over a large magnetic field range, in which case the in-plane WAL behavior could be identified as trivial anisotropic magnetoresistance (AMR). The in-plane quadratic MR has previously been observed in Bi_2_Te_3_^40^. However, to our knowledge, this is the first application of the Tkachov-Hankiewicz model to explain in-plane magnetoconductance data apart from the authors’ original culling of data available in the literature at the time of their derivation. As observed in Fig. 3, the data and model are in excellent agreement.

From the fits, values for the coherence length and effective surface state skin depth are extracted and shown in Fig. 3(d). The coherence length (skin depth) decreases (increases) with increasing Te concentration, with the WAL effect disappearing in Bi_2_Te_3_. The measured effective skin depth for each sample is on the order of the surface height roughness as seen in Fig. 1(a–d), which is much greater than the expected ~4 nm for the topological surface state skin depth from literature^42,43^. As this value is measure across a macroscopic sample, the effective surface depth is a convolution of the depth of the surface state and the roughness of the sample. Additionally, defects near the surface of the topological material can lead to an increase in the skin depth^44^. These samples are heavily electron doped ($n\approx {10}^{19}$ cm^−3^) which, along with the relative complexity of the band structure for Bi_2_Te_3_ relative to Bi_2_Se_3_, provides an explanation for the disappearance of the surface states. Bi_2_Se_3_ has a bulk bandgap of 0.3 eV, compared to 0.1 eV for Bi_2_Te_3_^45,46^. Additionally, the Dirac point in Bi_2_Te_3_ lies below the maximum of the bulk valence band while the Dirac point in Bi_2_Se_3_ lies above the bulk valence band. The larger band gap and less proximity of bulk states to the Dirac point allow Bi_2_Se_3_ to maintain its topological behavior over a wider range of dopings than Bi_2_Te_3_. In addition to the decrease in WAL signal, Te substitution is accompanied by an increase in the Hall mobility, owing to a Fermi energy firmly in the conduction band. Based on band structure calculations from the literature it is apparent that bulk bands are closer to the dirac point in Bi_2_Te_3_ than in Bi_2_Se_3_. Thus, the high carrier concentration in our samples has likely moved the Fermi level far enough into the bulk as to reduce the relative contribution of the surface states^18,23,45^. We expect the alloyed compositions to have band structures that are some linear combination of the Bi_2_Se_3_ and Bi_2_Te_3_ bands. From the measured effective penetration depth, we also expect some coupling of surface states on the top and bottom of the film.

From the fits, we see a coherent picture of the development of the topological states as Se is replaced by Te, with $\lambda$ being on the order of the sample roughness. This observation demonstrates the importance of film surface roughness to obtaining surface-pinned topological states that mix minimally with the bulk. At low temperature, there is a competition between the WAL from the surface states and a low field quadratic background. As the mobility at 3 K increases significantly in pure Bi_2_Te_3_ compared to the other samples, the relative size of the WAL cusp decreases leading to the near disappearance of WAL in Bi_2_Te_3_. A small cusp is visible, though overwhelmed by a quadratic background which precludes fitting to the Hikami-Larkin-Nagaoka model. The lack of angular dependence in the WAL signal from Bi_2_Se_3_ indicates a 3D origin, while the stronger theta-dependence in the alloyed samples indicates some 2D surface contribution.

In various other studies on Bi_2_Se_3_, a negative longitudinal magnetoresistance is observed when the field is applied along the current, often attributed to the chiral anomaly^47–49^. We do not observe this effect over the measured field range as the negative magnetoresistance effect is overwhelmed by the WAL at low fields. The in-plane WAL of the Tkachov-Hankiewicz model still occurs as it depends on the relative direction of the field to the surface, not the current direction. Thus, we consider the geometry that we used to be representative of the total angle-dependent field behavior at small fields.

**Figure 4:**
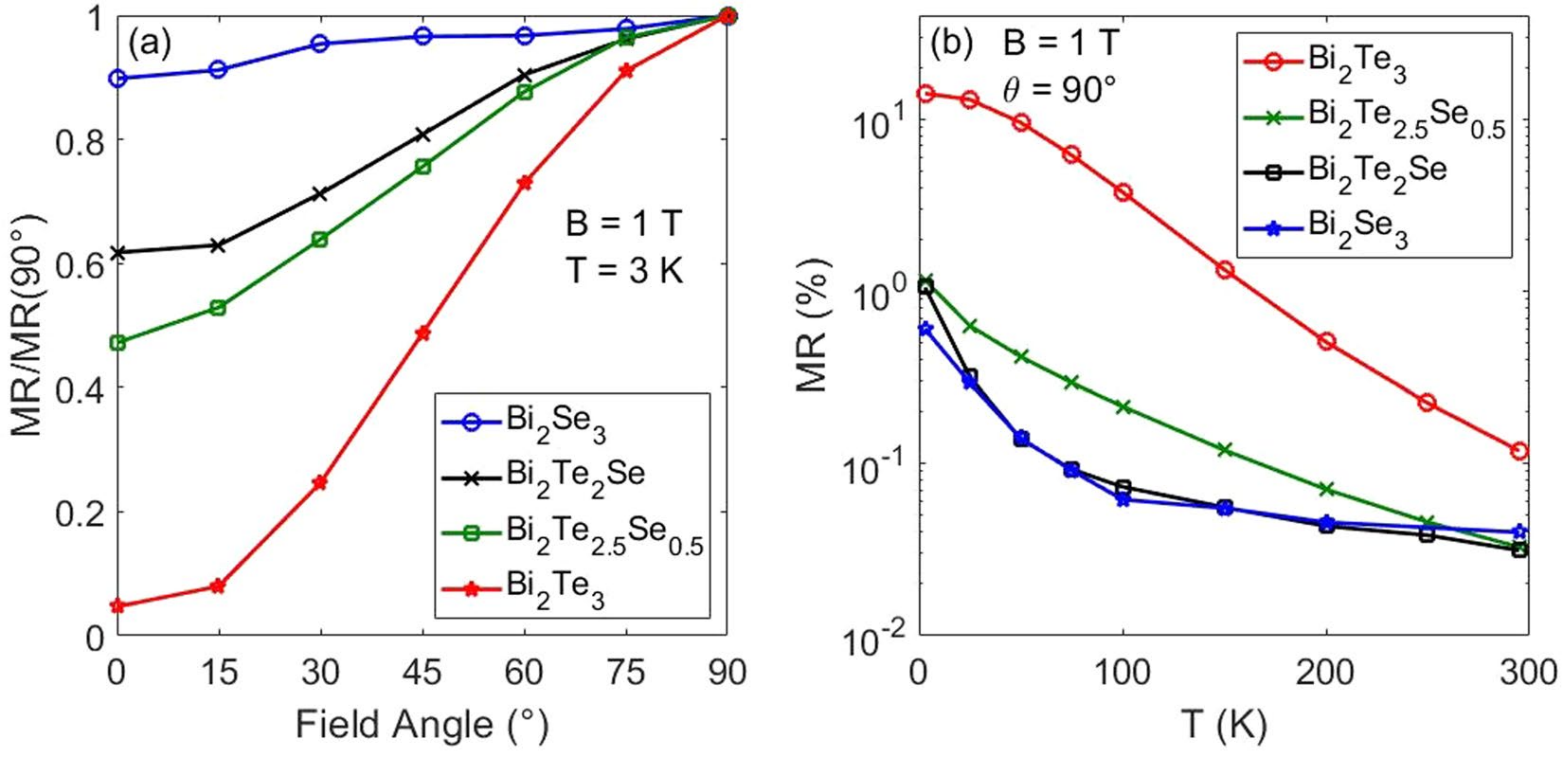
(**a**) MR vs. field angle, normalized to the value at θ = 90°. Bi_2_Te_3_ nearly follows a $\sin \,\theta$ dependence while Bi_2_Se_3_ is nearly constant with angle, with the Se, Te mixed samples decreasing between the extremes. (**b**) MR vs. Temperature. The Se containing samples decrease more quickly than the pure Bi_2_Te_3_ due to the rapid disappearance of WAL.

MR was also measured as the angle between field and sample is varied from 0° (in-plane) to 90° (out-of-plane). The change at B = 1 T as a function of angle and normalized to the value at 90° is plotted in Fig. 4(a) (see Supplemental Materials Fig. S2 for additional data). In conventional materials, anisotropic magnetoresistance arises from coupling to the out-of-plane magnetic field, leading to a dependence on B $\sin \,\theta$^50–53^. However, the increased θ-dependence of WAL for increasing Te contribution, up to x = 2.5, suggests a stronger surface contribution in Bi_2_Te_2.5_Se_0.5_ than in Bi_2_Se_3_.

Figure 4(b) shows the temperature dependence of the MR. As expected, the change in MR decreases with increasing temperature. The samples that show WAL have a cusp that broadens with T and develops into fully quadratic MR by 100 K. The MR at 1 T is plotted vs. temperature in Fig. 4(b). The rapid decrease in MR in the Bi_2_Se_3_ and Bi_2_Te_2_Se samples is due to the disappearance of WAL, while Bi_2_Te_3_ decreases steadily from the steadily decreasing mobility. The WAL decrease follows nearly the same divergent dependence in T as does $\Delta \sigma (B)$ due to the temperature dependence of the coherence length ($L\, \sim \,{T}^{-1}$) and the divergence of $\psi (x)$. Bi_2_Te_2.5_Se_0.5_ decreases slower than the other Se-containing samples due to a relatively stronger background MR.

## Conclusion

In conclusion, we have demonstrated non-trivial AMR in Bi_2_Se_3_ arising from a non-zero skin depth of the topological surface states. As the Se is substituted for Te, the surface states are overshadowed by bulk conduction, leading to more conventional behavior in Bi_2_Te_3_. This results in distinctive weak antilocalization and non-trivial anisotropic magnetoresistance in Se-containing samples which does not appear in Bi_2_Te_3_. The Tkachov-Hankiewicz coupled with the Hikami-Larkin-Nagaoka model provides a straightforward means of measuring both the coherence length and characterizing the quality of the surface states through the effective skin depth. We have also demonstrated that careful investigation of the low field in-plane MR in topological materials can provide valuable insight into surface states and sample quality, information that will prove vital to best utilizing topological surface states and optimizing film growths.

## Supplementary information

Supplementary information.

## References

[1] Hasan MZ, Kane CL (2010). Colloquium: Topological insulators. Rev. Mod. Phys..

[2] Qi X-L, Zhang S-C (2010). The quantum spin Hall effect and topological insulators. Phys. Today.

[3] Li CH (2014). Electrical detection of charge-current-induced spin polarization due to spin-momentum locking in Bi_2_Se_3_. Nat. Nanotechnol..

[4] Liu L (2015). Spin-polarized tunneling study of spin-momentum locking in topological insulators. Phys. Rev. B.

[5] Narendra N, Norouzzadeh P, Vashaee D, Kim KW (2017). Doping induced enhanced density of states in bismuth telluride. Appl. Phys. Lett..

[6] Butch NP (2010). Strong surface scattering in ultrahigh-mobility Bi_2_Se_3_ topological insulator crystals. Phys. Rev. B.

[7] Neupane M (2014). Observation of a three-dimensional topological Dirac semimetal phase in high-mobility Cd3As2. Nat. Commun..

[8] Shekhar C (2015). Extremely large magnetoresistance and ultrahigh mobility in the topological Weyl semimetal candidate NbP. Nat. Phys..

[9] Xiu F (2011). Manipulating surface states in topological insulator nanoribbons. Nat. Nanotechnol..

[10] Fan Y (2014). Magnetization switching through giant spin-orbit torque in a magnetically doped topological insulator heterostructure. Nat. Mater..

[11] Semenov YG, Li X, Kim KW (2012). Tunable photogalvanic effect on topological insulator surfaces via proximity interactions. Phys. Rev. B.

[12] Duan X, Li X-L, Li X, Semenov YG, Kim KW (2015). Highly efficient conductance control in a topological insulator based magnetoelectric transistor. J. Appl. Phys..

[13] Pesin D, MacDonald AH (2012). Spintronics and pseudospintronics in graphene and topological insulators. Nat. Mater..

[14] Jing Y (2016). Weak antilocalization and electron-electron interaction in coupled multiple-channel transport in a Bi_2_Se_3_ thin film. Nanoscale.

[15] Kong D (2010). Few-layer nanoplates of Bi_2_Se_3_ and Bi_2_Te_3_ with highly tunable chemical potential. Nano Lett..

[16] Steinberg H, Laloë J-B, Fatemi V, Moodera JS, Jarillo-Herrero P (2011). Electrically tunable surface-to-bulk coherent coupling in topological insulator thin films. Phys. Rev. B.

[17] Steinberg H, Gardner DR, Lee YS, Jarillo-Herrero P (2010). Surface state transport and ambipolar electric field effect in Bi 2Se3 nanodevices. Nano Lett..

[18] Chen YL (2009). Experimental Realization of a Three-Dimensional Topological Insulator, Bi_2_Te_3_. Science.

[19] Chang C-Z (2015). High-precision realization of robust quantum anomalous Hall state in a hard ferromagnetic topological insulator. Nat. Mater..

[20] He L (2011). Epitaxial growth of Bi_2_Se_3_ topological insulator thin films on Si (111). J. Appl. Phys..

[21] Ota JR, Roy P, Srivastava SK, Popovitz-Biro R, Tenne R (2006). A simple hydrothermal method for the growth of Bi_2_Se_3_ nanorods. Nanotechnology.

[22] Zeng Z (2013). Molecular beam epitaxial growth of Bi_2_Te_3_ and Sb_2_Te_3_ topological insulators on GaAs (111) substrates: a potential route to fabricate topological insulator p-n junction. AIP Adv..

[23] Hor YS, Checkelsky JG, Qu D, Ong NP, Cava RJ (2011). Superconductivity and non-metallicity induced by doping the topological insulators Bi_2_Se_3_ and Bi_2_Te_3_. J. Phys. Chem. Solids.

[24] Kushwaha SK (2016). Sn-doped Bi1.1Sb0.9Te2S bulk crystal topological insulator with excellent properties. Nat. Commun..

[25] Kapustin AA, Stolyarov VS, Bozhko SI, Borisenko DN, Kolesnikov NN (2015). Surface origin of quasi-2D Shubnikov–de Haas oscillations in Bi_2_Te_2_Se. J. Exp. Theor. Phys..

[26] Liu X (2012). Characterization of Bi_2_Te_3_ and Bi_2_Se_3_ topological insulators grown by MBE on (001) GaAs substrates. J. Vac. Sci. Technol. B.

[27] Levy I, Garcia TA, Shafique S, Tamargo MC (2018). Reduced twinning and surface roughness of Bi_2_Se_3_ and Bi_2_Te_3_ layers grown by molecular beam epitaxy on sapphire substrates. J. Vac. Sci. Technol. B.

[28] Wiedmann S (2016). Anisotropic and strong negative magnetoresistance in the three-dimensional topological insulator Bi_2_Se_3_. Phys. Rev. B.

[29] Long D, Myers J (1959). Ionized-Impurity Scattering Mobility of Electrons in Silicon. Phys. Rev..

[30] Chattopadhyay D, Queisser HJ (1981). Electron scattering by ionized impurities in semiconductors. Rev. Mod. Phys..

[31] Hikami S, Larkin AI, Nagaoka Y (1980). Spin-Orbit Interaction and Magnetoresistance in the Two Dimensional Random System. Prog. Theor. Phys..

[32] Bergmann G (1982). Measurement of the Magnetic Scattering Time by Weak Localization. Phys. Rev. Lett..

[33] Assaf BA (2014). Quantum coherent transport in SnTe topological crystalline insulator thin films. Appl. Phys. Lett..

[34] Bao L (2012). Weak Anti-localization and Quantum Oscillations of Surface States in Topological Insulator Bi_2_Se_2_Te. Sci. Rep..

[35] Johnson HG, Bennett SP, Barua R, Lewis LH, Heiman D (2010). Universal properties of linear magnetoresistance in strongly disordered MnAs-GaAs composite semiconductors. Phys. Rev. B.

[36] Abrikosov AA (2000). Quantum linear magnetoresistance. Europhys. Lett..

[37] Assaf BA (2013). Linear magnetoresistance in topological insulator thin films: Quantum phase coherence effects at high temperatures. Appl. Phys. Lett..

[38] Parish MM, Littlewood PB (2005). Classical magnetotransport of inhomogeneous conductors. Phys. Rev. B.

[39] Friedman AL (2010). Quantum linear magnetoresistance in multilayer epitaxial graphene. Nano Lett..

[40] He H-T (2011). Impurity Effect on Weak Antilocalization in the Topological Insulator Bi_2_Te_3_. Phys. Rev. Lett..

[41] Tkachov G, Hankiewicz EM (2011). Weak antilocalization in HgTe quantum wells and topological surface states: Massive versus massless Dirac fermions. Phys. Rev. B.

[42] Zhang F, Kane CL, Mele EJ (2012). Surface states of topological insulators. Phys. Rev. B.

[43] Vidal F (2013). Photon energy dependence of circular dichroism in angle-resolved photoemission spectroscopy of Bi_2_Se_3_ Dirac states. Phys. Rev. B.

[44] Sacksteder V, Ohtsuki T, Kobayashi K (2015). Modification and control of topological insulator surface states using surface disorder. Phys. Rev. Appl..

[45] Zhang H (2009). Topological insulators in Bi_2_Se_3_, Bi_2_Te_3_ and Sb_2_Te_3_ with a single Dirac cone on the surface. Nat. Phys..

[46] Yazyev OV, Moore JE, Louie SG (2010). Spin Polarization and Transport of Surface States in the Topological Insulators Bi_2_Se_3_ and Bi_2_Te_3_ from First Principles. Phys. Rev. Lett..

[47] He HT (2013). Disorder-induced linear magnetoresistance in (221) topological insulator Bi_2_Se_3_ films. Appl. Phys. Lett..

[48] Wang J (2012). Anomalous anisotropic magnetoresistance in topological insulator films. Nano Res..

[49] Assaf BA (2017). Negative Longitudinal Magnetoresistance from the Anomalous N=0 Landau Level in Topological Materials. Phys. Rev. Lett..

[50] Kandala A, Richardella A, Kempinger S, Liu C-X, Samarth N (2015). Giant anisotropic magnetoresistance in a quantum anomalous Hall insulator. Nat. Commun..

[51] Vašek P (2010). Anisotropic magnetoresistance of GaMnAs ferromagnetic semiconductors. J. Supercond. Nov. Magn..

[52] Huang H (2018). Giant anisotropic magnetoresistance and planar Hall effect in Sr_0.06_ Bi_2_Se_3_. Appl. Phys. Lett..

[53] Li H, Wang H-W, He H, Wang J, Shen S-Q (2018). Giant anisotropic magnetoresistance and planar Hall effect in the Dirac semimetal Cd_3_As_2_. Phys. Rev. B.

