# Supplementary material for: Weak Antilocalization and Anisotropic Magnetoresistance as a Probe of Surface States in Topological Bi2TexSe3−x Thin Films
Source: Sci Rep. 2020 Mar 16;10:4845. doi: 10.1038/s41598-020-61672-1 (PMC7076004; doi:10.1038/s41598-020-61672-1)
Supplement: Supplementary file 1 — Supplementary information. [file 41598_2020_61672_MOESM1_ESM.docx]

**Weak Antilocalization and Anisotropic Magnetoresistance as a Probe of Surface States in Topological Bi_2_Te_x_Se_3-x_** **Thin Films**

Gregory M. Stephen^1^, Owen. A Vail^2^, Jiwei Lu^3^, William A. Beck^2^, Patrick J. Taylor^2^, Adam L. Friedman^1^

^1^*Laboratory for Physical Sciences, 8050 Greenmead Dr., College Park, MD 20740*

^2^*Army Research Laboratory, 2800 Powder Mill Rd., Adelphi, MD 20783*

*^3^Department of Materials Science and Engineering, University of Virginia, Charlottesville, VA 22904*

**Chiral Anomaly:**

The chiral anomaly often present in topological materials appears as a negative magnetoresistance (NMR) when the field is applied along the current.^1^ **Fig. S1** shows the in-plane MR with Magnetic field applied both parallel and perpendicular to the current *I*. The WAL cusp is identical for the two directions for B < 0.5 T, with a negative deviation up to 1 T for $B\parallel I$, though continually increasing with B. The deviation is a result of the added NMR due to the chiral anomaly, though the WAL background is strong enough to dominate over the measured field range. This indicates that the chiral anomaly, while present, is primarily a high-field effect. Thus, it is not relevant for the analysis presented in this study.


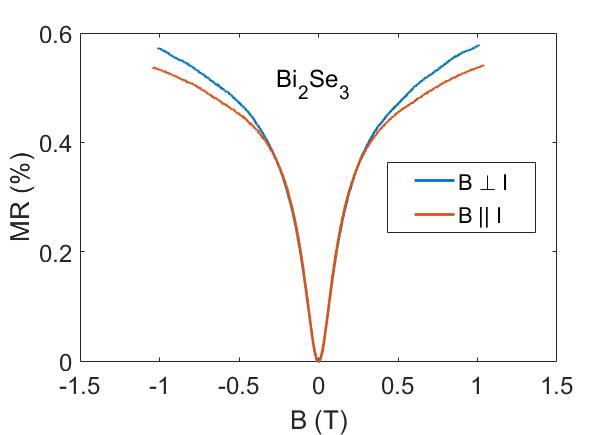


Figure S1: In-plane MR with Field aligned parallel and perpendicular to the current. The negative magnetoresistance associated with the chiral anomaly is not observed, though the faster downturn of the parallel data is likely a result of its onset.

**Magnetoresistance:**

**Fig. S2** shows the MR plotted versus $B\sin\theta$ to further demonstrate the non-trivial anisotropy in the magnetoresistance. For trivial AMR where the angular dependence is due to coupling to the perpendicular field, the curves should overlap, as is seen in Bi_2_Te_3_. For Bi_2_Se_3_, we see that not only is there no overlap between the different field angles, but the maximum change is nearly constant from 15° (blue) to 90° (black). 0° data is not plotted as sin 0 = 0. The Se, Te mixed samples lie in between the two extremes.


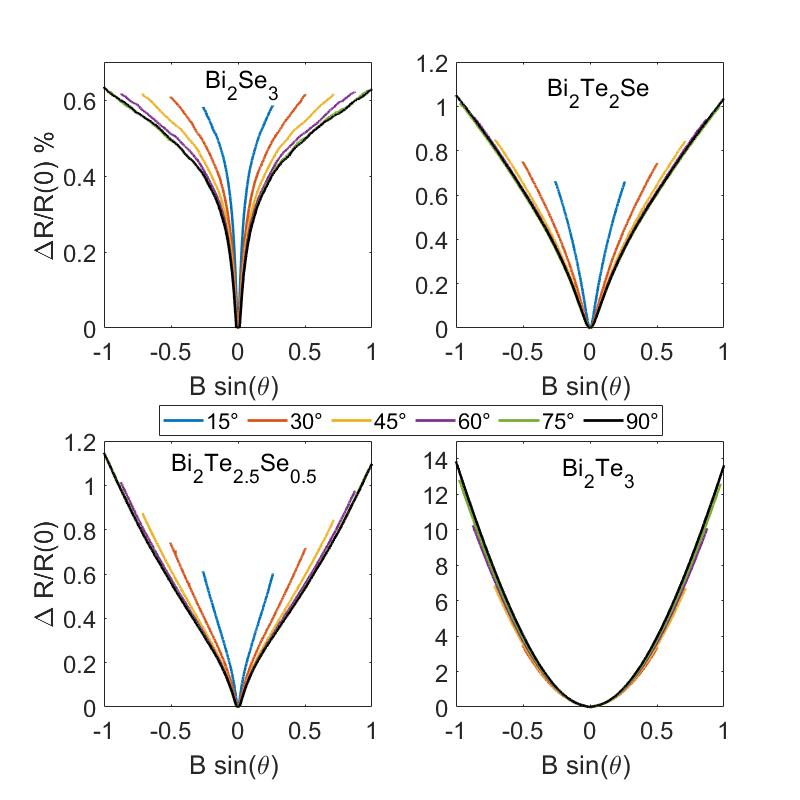


Figure S2: MR plotted against $B\sin\theta$demonstrating the non-trivial AMR for the Se containing samples.

**Fig. S3** shows the full field sweeps at T ranging from 3 to 295 K. In Bi_2_Se_3_ the WAL broadens with increasing temperature, disappearing by 100 K and giving way to classical quadratic MR. This broadening is apparent in the alloyed samples as well, though the WAL vanishes by 25 K in both. Bi_2_Te_3_ shows quadratic MR for all temperatures.


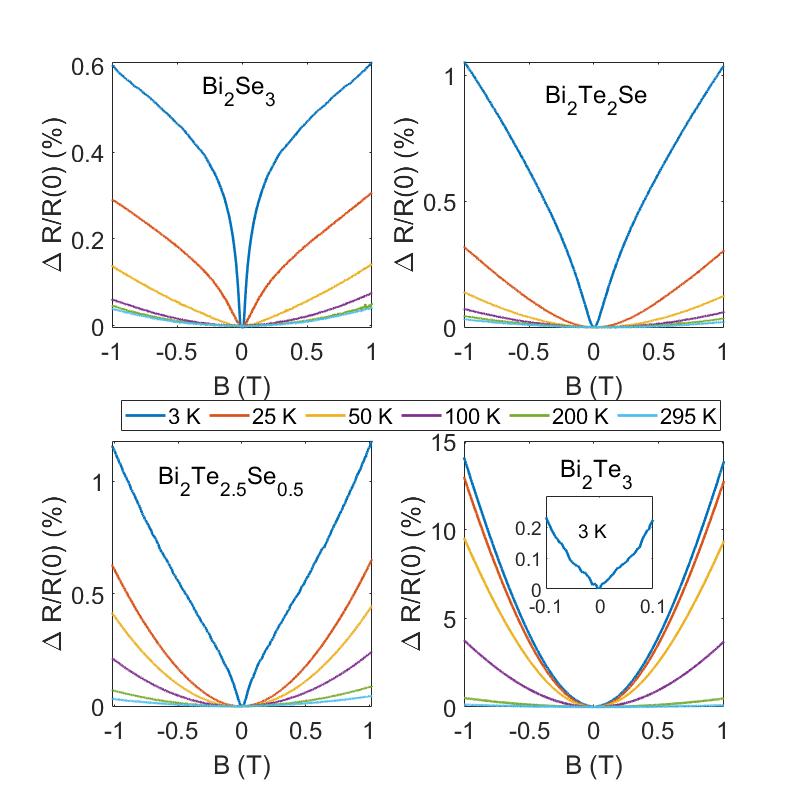


Figure S3: Temperature dependent MR

MR measurements up to 9 T at T = 4 K are displayed in **Fig. S4**. High field data were taken using a Cryomagnetics liquid helium cryostat using a 9 Tesla superconducting magnet at 4.2 K. Electrical measurements were taken using standard lock-in techniques at low frequency for both positive and negative magnetic field sweeps. The WAL in the Se-containing samples is still apparent, though with the background MR more clearly shown. The two alloyed samples show linear MR up to 9 T, with Bi_2_Se_3_ showing quadratic MR out to near 6 T, becoming linear at higher fields. As high field behavior was not the primary concern in this study, these data were only used to subtract out background behavior and are presented here as reference. A weak WAL cusp at 3 K in Bi_2_Te_3_ is shown as an inset. The strong quadratic background precludes fitting to either WAL model.


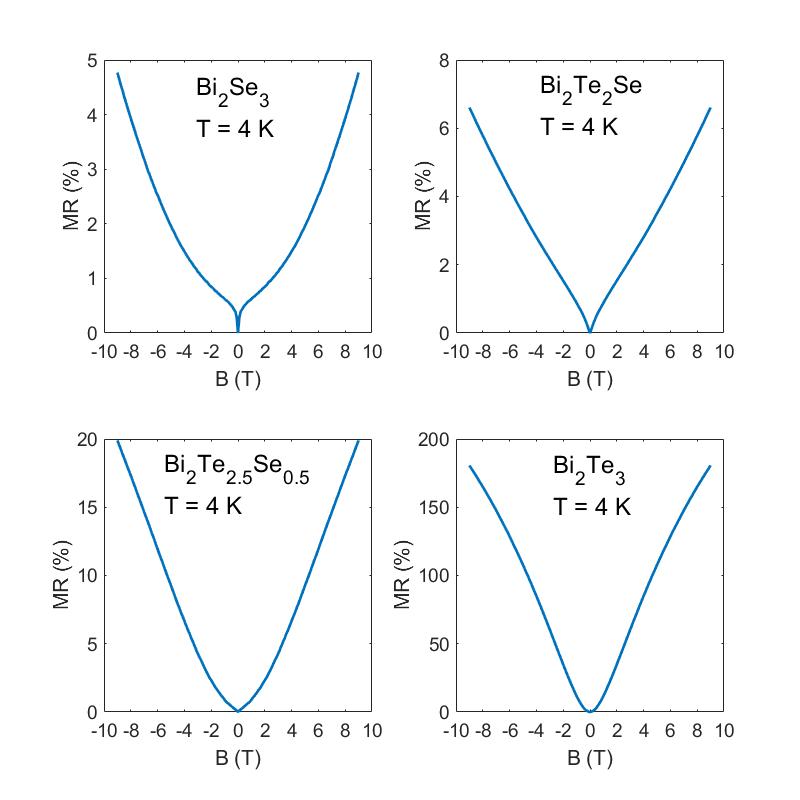


Figure S4: MR up to 9T for each sample at T =4 K.

**X-Ray Characterization:**

High-resolution x-ray diffraction was used to determine the epitaxial quality as well as alloy composition for the Bi_2_Te_x_Se_3-x_ **Fig. S5** shows the obtained x-ray spectra for the samples of this study showing the change in Bragg angle with alloy. For all samples, large angle scans showed that there were well-defined peaks that are exactly indexed to a trigonal R$\bar{3}$m space group which confirms highly oriented, excellent quality epitaxial growth. The peak intensity remains roughly constant throughout because the thickness was constant at 50 nm. The relative change in position of the (0015) reflection was used, assuming Vegardian behavior, to quantify the alloy composition.


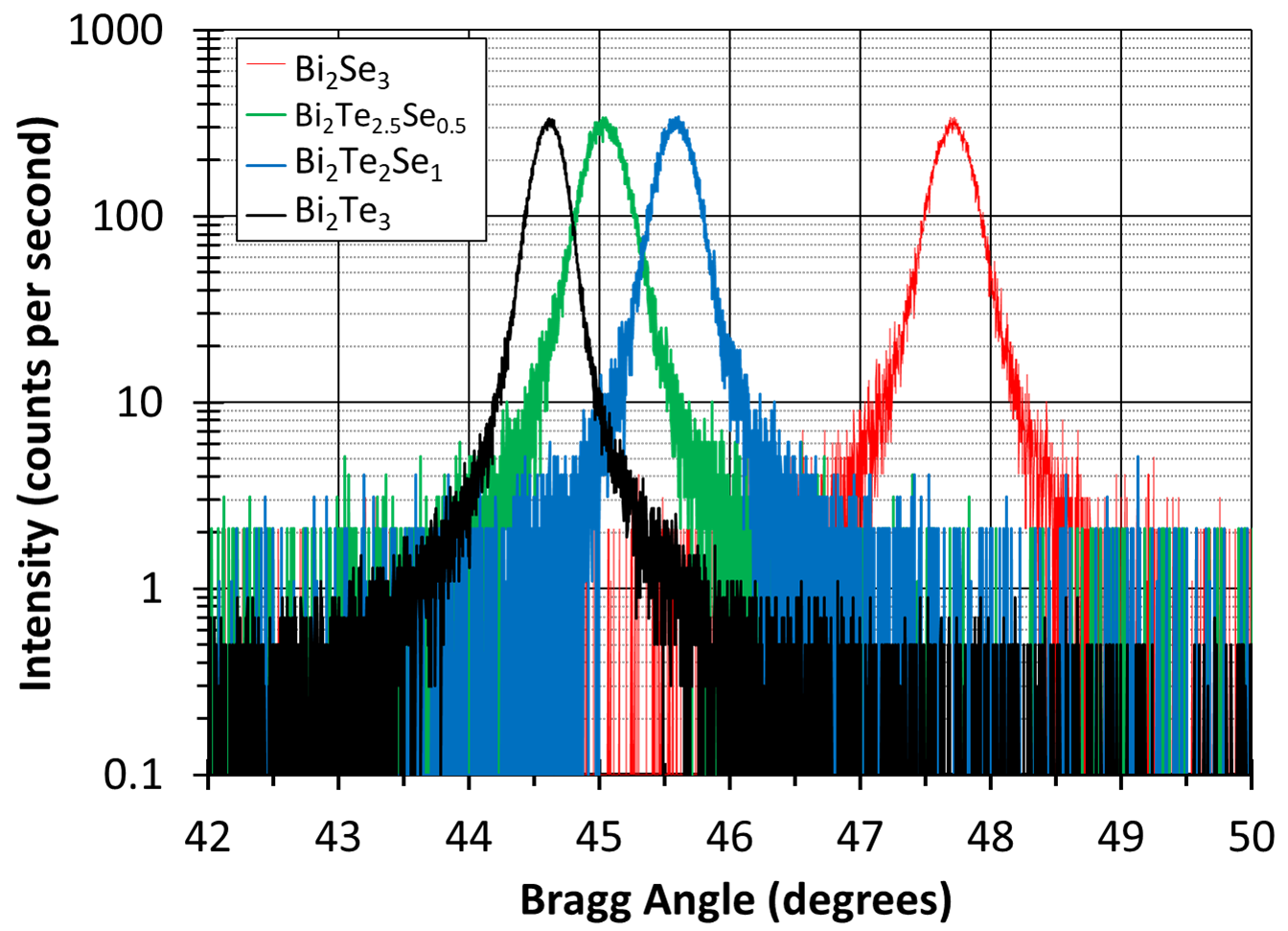


Figure S5: X-ray diffraction spectra from Bi_2_Te_x_Se_3-x_

The MBE of Bi_2_Te_x_ Se_(3-x)_ materials on (001) GaAs proceeded in a layer-by-layer mode, and adopted an <0001> growth direction. The RHEED patterns showed a brilliant (1x1) surface reconstruction after a few nanometers of growth as shown in the obtained RHEED pattern.


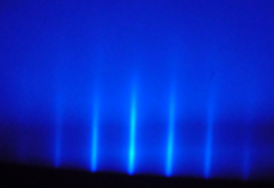


*Figure S6: RHEED image showing (1X1) surface reconstruction during MBE of Bi_2_Te_x_ Se_(3-x)_*

TEM examination of the Bi_2_Te_x_ Se_(3-x)_/GaAs heterostructure revealed an extremely sharp epitaxial interface. Despite the somewhat large lattice misfit and different crystal structure, this MBE approach routinely yields robust high-quality Bi_2_Te_x_ Se_(3-x)_ materials that remain largely devoid of structural defects.


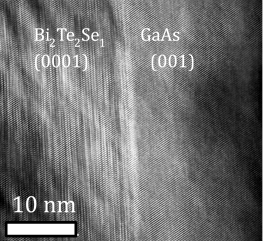


*Figure S7: TEM images showing heteroepitaxial interface between Bi_2_Te_x_ Se_(3-x)_ and GaAs.*

**Additional Transport Data**


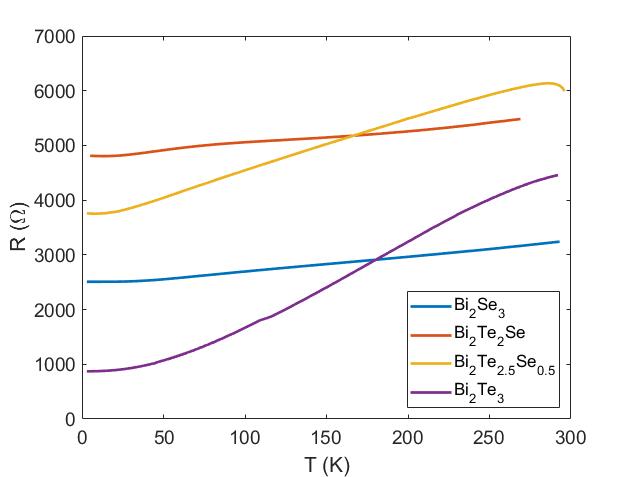


Figure S8: Resistance versus Temperature for all four samples

1. Li, Y. *et al.* Negative magnetoresistance in Weyl semimetals NbAs and NbP: Intrinsic chiral anomaly and extrinsic effects. *Front. Phys.* **12**, 127205 (2017).
